# Supplementary material for: Integrated model of the vertebrate augmin complex
Source: Nat Commun. 2023 Apr 13;14:2072. doi: 10.1038/s41467-023-37519-4 (PMC10102177; doi:10.1038/s41467-023-37519-4)
Supplement: Supplementary file 1 — Supplementary Info [file 41467_2023_37519_MOESM1_ESM.pdf]

SUPPLEMENTARY INFORMATION

for

Integrated Model of the Vertebrate Augmin Complex

Sophie M Travis[1], Brian P Mahon[1,2], Wei Huang[3], Meisheng Ma[4,5], Michael J Rale[1,6], Jodi Kraus[1], Derek J Taylor[3], Rui Zhang[4*], Sabine Petry[1*]

[1]Department of Molecular Biology, Princeton University, Princeton, NJ, USA
[2]Present address: Department of Structural Biology, Bristol Myers Squibb, Princeton, NJ, USA
[3]Department of Pharmacology, Case Western Reserve University, Cleveland, OH, USA
[4]Department of Biochemistry and Molecular Biophysics, Washington University in St. Louis, School of Medicine, St. Louis, MO, USA
[5]Present address: Department of Histology and Embryology, School of Basic Medicine, Tongji Medical College, Huazhong University of Science and Technology, Wuhan, Hubei, China.
[6]Present address: Department of Cell Biology, Harvard Medical School, Boston, MA, USA
These authors contributed equally: Sophie M Travis, Brian P Mahon
Correspondence and requests for materials should be addressed to Rui Zhang (email: zhangrui@wustl.edu) or Sabine Petry (email: spetry@princeton.edu)

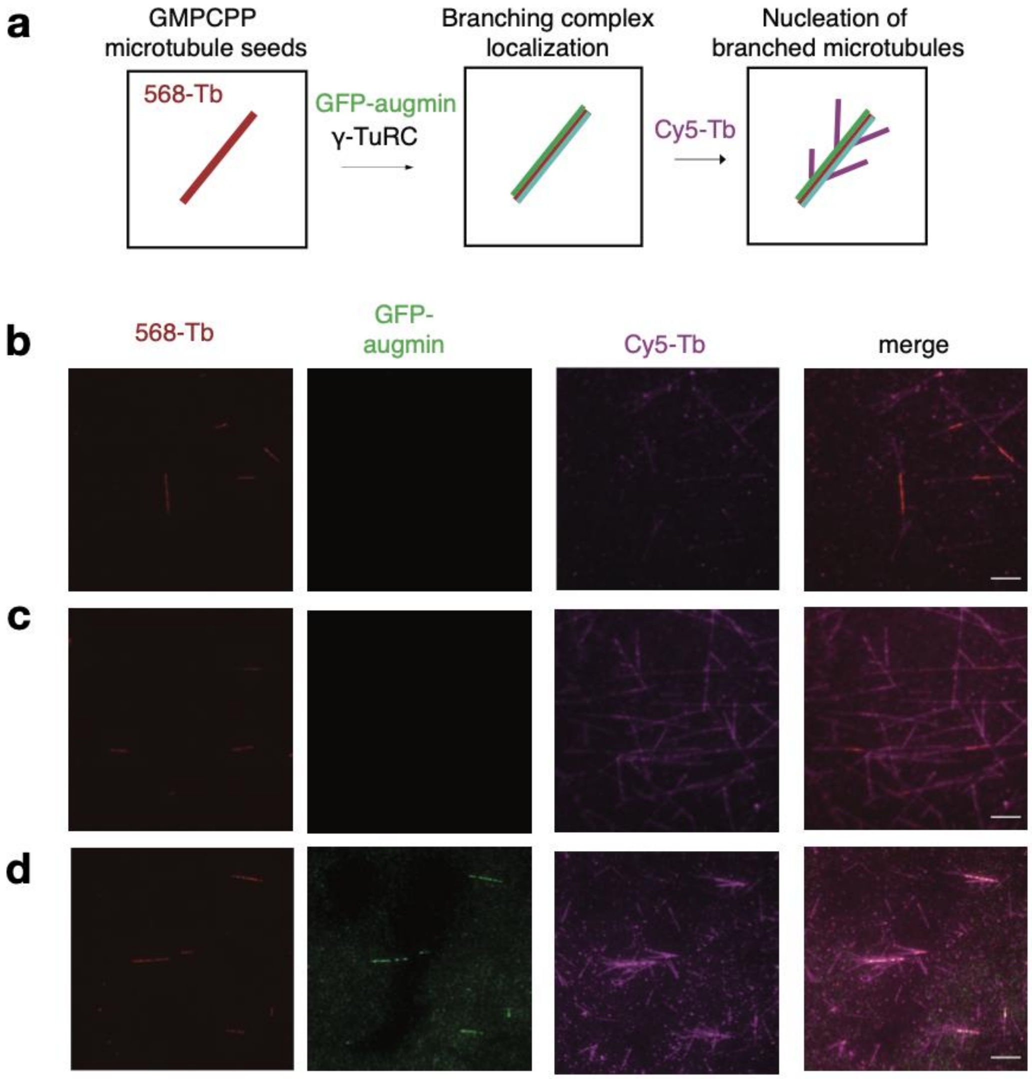

<u>Supplementary Figure 1: In vitro reconstitution of branching MT nucleation via TIRF microscopy</u>
a. Schematic of in vitro branching reconstitution. Single cycled, GMPCPP stabilized MT seeds are attached to biotin-PEG functionalized glass cover slips via neutravidin and incorporation of biotin into the MT seeds. Following this, the branching factor complex containing augmin and γ-TuRC are bound to MT seeds. Finally, Cy5 labeled soluble tubulin was flowed into the reaction chambers to nucleate new MTs.
b. Representative TIRF microscopy images from in vitro branching reconstitution performed with buffer only. MT seeds are visualized with Alexa-568 (red, first column), augmin is visualized by GFP labeling (green, 2nd column), and soluble tubulin is visualized by Cy5 labeling (3rd column). Merged images are shown in the last column. Only branching machinery containing augmin and γ-TuRC can effectively recruit soluble tubulin to the MT and initiate new branched MTs. Scale bars indicate 5 μM. Two technical replicates were performed.
c. Representative TIRF microscopy images from in vitro branching reconstitution performed with γ-TuRC only. Reaction was performed as in (b).
d. Representative TIRF microscopy images from in vitro branching reconstitution performed with augmin and γ-TuRC. Reaction was performed as in (b).

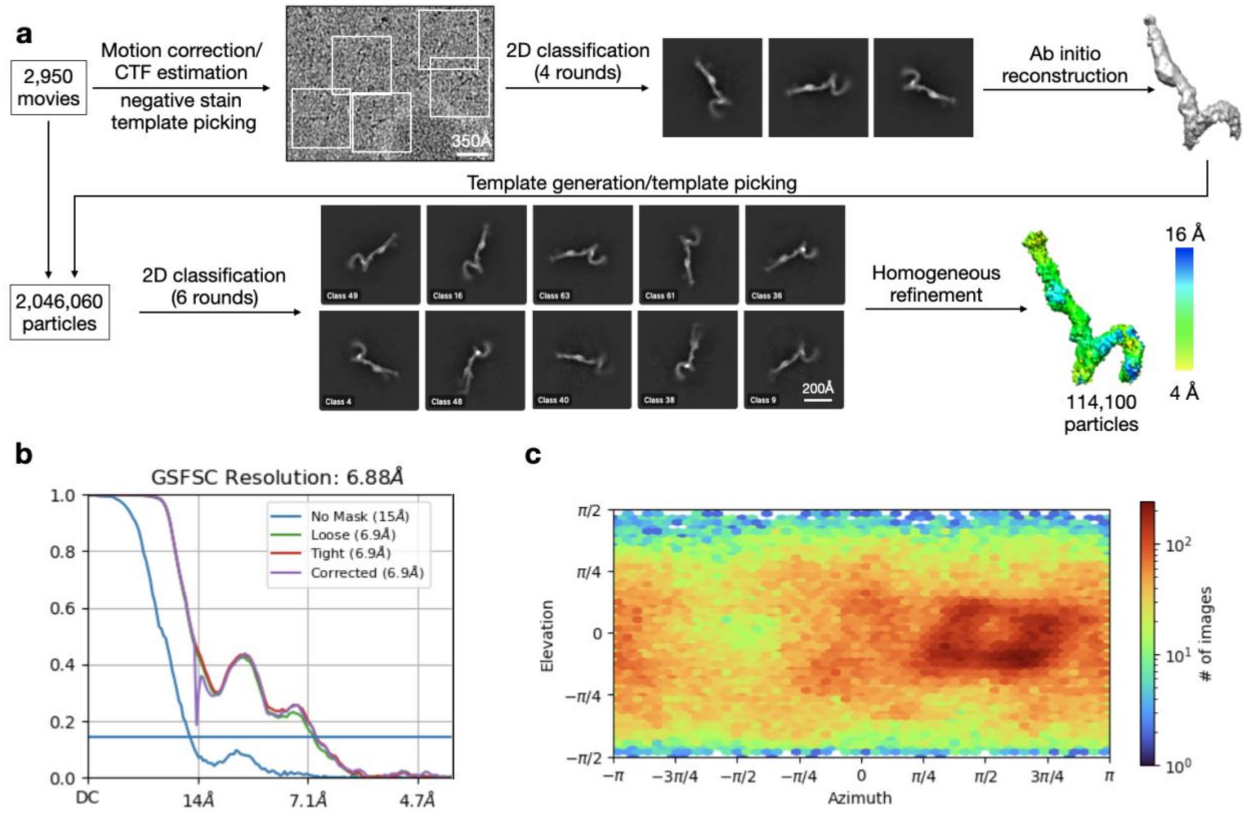

<u>Supplementary Figure 2: Workflow of cryo-EM data processing and map statistics</u>

a. The workflow used, in CryoSPARC, to correct movies, pick particles, classify particles, and refine orientations, leading to a final map using 114,100 particles. Final map is colored by local resolution, where high resolution sections are yellow, moderate resolution are green, and low resolution are blue.

b. Gold-standard FSC curves for determining cryo-EM map resolution are displayed, showing a resolution cut-off of 6.9 Å.

c. Viewing direction distribution of particles in final map, showing a preferred orientation cluster at π/2.

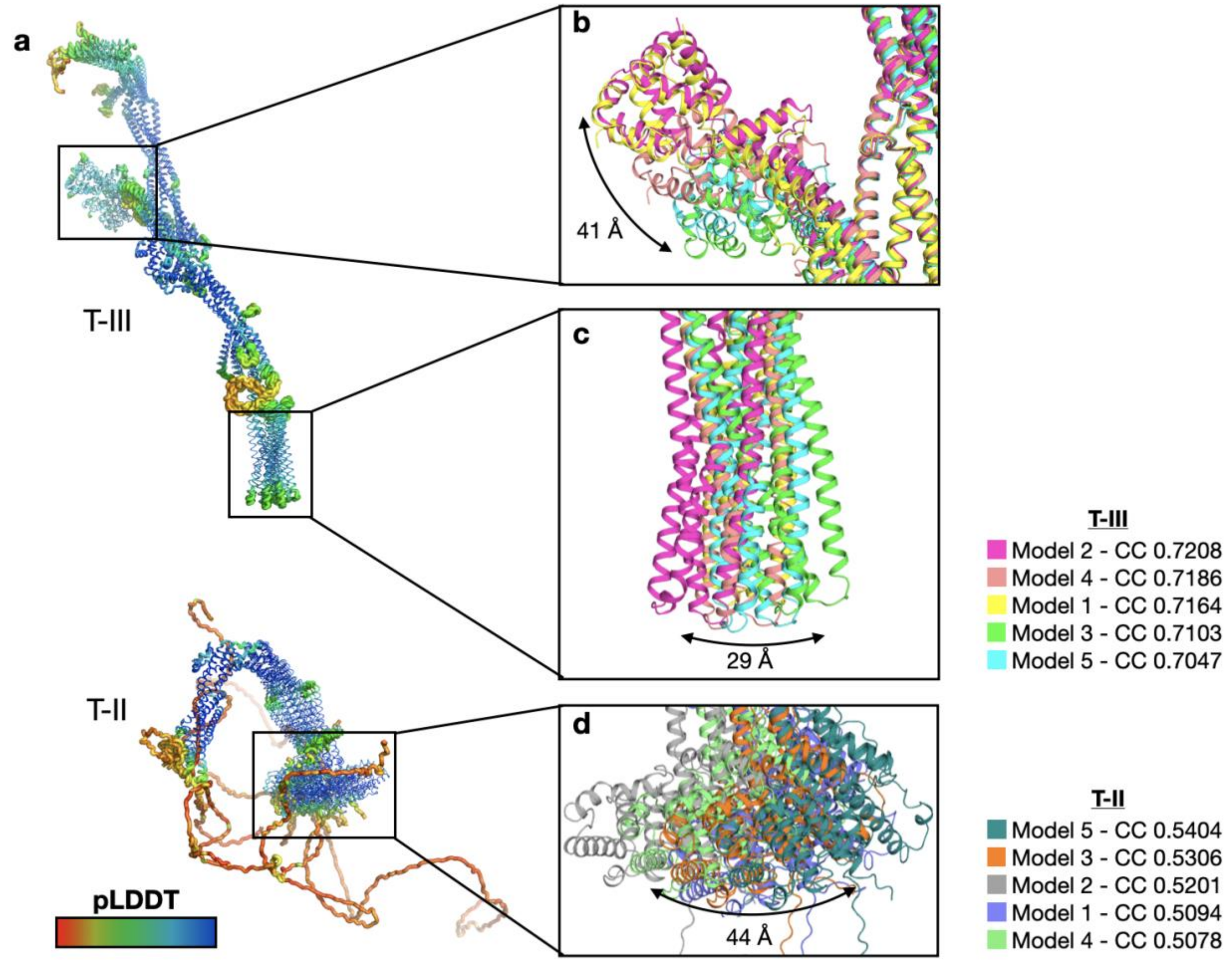

Supplementary Figure 3: Modeling augmin T-II and T-III structures using AlphaFold2-Multimer

a. Five models of *X. laevis* T-III and T-II, independently determined by AlphaFold2-Multimer, were superimposed, and colored by their coordinate confidence, or predicted local distance displacement test (pLDDT), where red corresponds to low pLDDT and low confidence, and blue corresponds to high pLDDT and high confidence. The low confidence unstructured sequence within T-II depicts the MTBD of Haus8, which differs dramatically between the five models.

b-d. High confidence (high pLDDT) regions of the T-III second leg (b), T-III base (c), and T-II globular domains (d) are enlarged to show how the five independent models differ in conformation between these three regions. The maximum distance between the models is calculated and shown beneath the arrow demonstrating the direction of motion. At right, the cross-correlation between each of the five models (as a theoretical 6.9 Å resolution surface) and the corresponding region of the augmin cryo-EM map, is shown for each model, ranking the models in terms of their fit within the map as calculated by ChimeraX fitmap.

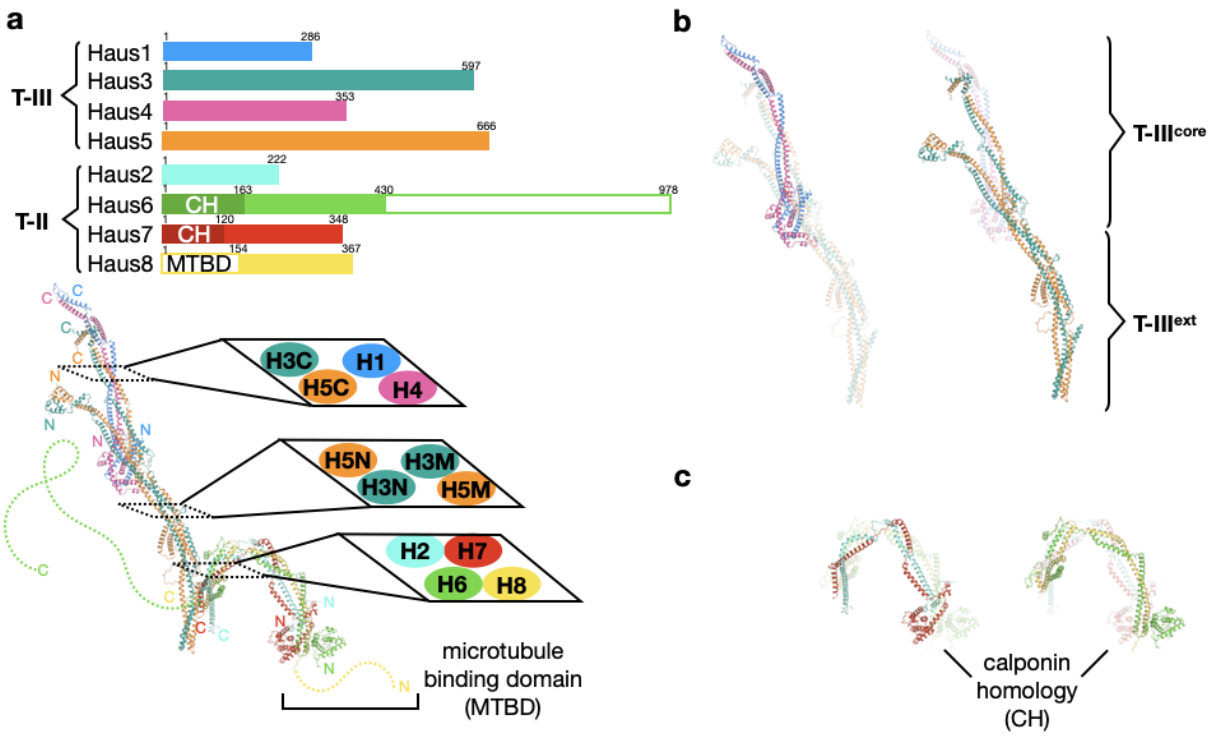

Supplementary Figure 4: Architecture of the augmin complex

a. Above, cartoon showing size of the eight augmin subunits and annotating all identified domains. In addition, the C-terminal region of Haus6 that was removed from our construct is indicated to scale. Below, integrated model of the *X. laevis* augmin complex, highlighting predicted locations of N- and C-termini of all eight subunits, as well as the MT binding site present within the extreme disordered N-terminus of Haus8. Inset cross-sections through 4-helix bundles in T-II, T-III^{core}, and T-III^{ext} show the architecture of the tetrameric parallel bundles within T-II and T-III^{core}, as well as the antiparallel extended hairpin comprising T-III^{ext}. Three helices derived from Haus3 appear in cross section, from the N-terminus (H3N) through the middle (H3M) to the C-terminus (H3C). Similarly, Haus5 appears in three cross-sections: N-terminal (H5N), middle (H5M), and C-terminal (H5C).

b. T-III is a dimer comprised of two dimers: highlighted at left, the paralogs Haus1 (blue) and Haus4 (pink); highlighted at right, the much larger paralogs Haus3 (green) and Haus5 (orange).

c. T-II is a dimer comprised of two dimers: highlighted at left, Haus6 (green) and Haus8 (yellow); highlighted at right, Haus2 (cyan) and Haus7 (red). Haus6 and Haus7 both contain calponin homology domains at their N-termini, marking the two as paralogs of one another.

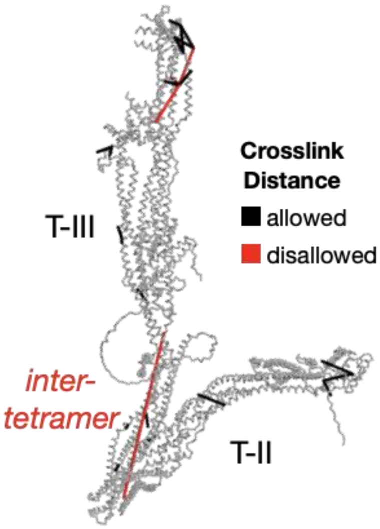

Supplementary Figure 5: Model validation of *D. melanogaster* augmin using cross-linking mass spectrometry

Twenty-six high-confidence BS3 inter-subunit primary amine crosslinks that had previously been experimentally determined for *D. melanogaster* augmin were mapped to the integrated *D. melanogaster* augmin complex model. Inter-nitrogen distances within the allowed spacer arm length of 24 Å are indicated in black, whereas disallowed crosslinks of more than 24 Å are indicated in red. 22 out of 26 crosslinks are allowed. Crosslinking mass spectrometry data was derived from[1]. Only crosslinks observed more than once are displayed.

a

	Species	Haus6
Animalia	*Brugia malayi*	N/A
	Caenorhabditis elegans	N/A
	Trichinella spiralis	XP_003379630.1
	Drosophila melanogaster	NP_651671.1
	Apis mellifera	NP_001242967.1
	Ixodes	XP_002412271.3
	Strongylocentrotus purpuratus	XP_030832423.1
	Xenopus laevis	XP_041438316.1
	Homo sapiens	NP_060115.3
	Trichoplax adhaerens	XP_002111058.1
	Monosiga brevicollis	N/A
Fungi	*Saccharomyces cerevisiae*	N/A
	Pichia	N/A
	Yarrowia lipolytica	N/A
	Emericella nidulans	XP_663791.1
	Schizosaccharomyces pombe	N/A
	Laccaria bicolor	XP_001886486.1
	Ustilago maydis	N/A
	Phycomyces blakesleeanus	XP_018285460.1
	Batrachochytrium dendrobatidis	XP_006679912.1
Amoebozoa	*Dictyostelium discoideum*	N/A
	Acanthamoeba castellani	XP_004336105.1
SAR	*Theileria equi*	N/A
	Plasmodium falciparum	N/A
	Toxoplasma gondii	N/A
	Tetrahymena thermophila	XP_001008516.1
	Paramecium	N/A
	Phaeodactylum tricornutum	N/A
	Thalassiosira oceanica	N/A
	Aureococcus anophagefferens	N/A
	Phytophthora infestans	N/A
Plantae	*Oryza sativa*	XP_015625562.1
	Arabidopsis thaliana	NP_568585.1
	Selaginella moellendorfii	XP_024525773.1
	Physcomitrella patens	XP_024393130.1
	Micromonas pusilla	N/A
	Ostreococcus tauri	N/A
	Chlorella variabilis	XP_005842913.1
	Volvox carteri	XP_002957730.1
	Chlamydomonas reinhardtii	XP_042915056.1
Discoba	*Trypanosoma brucei*	N/A
	Leishmania donovani	N/A
	Euglena gracilis	N/A
	Naegleria fowleri	XP_044564931.1

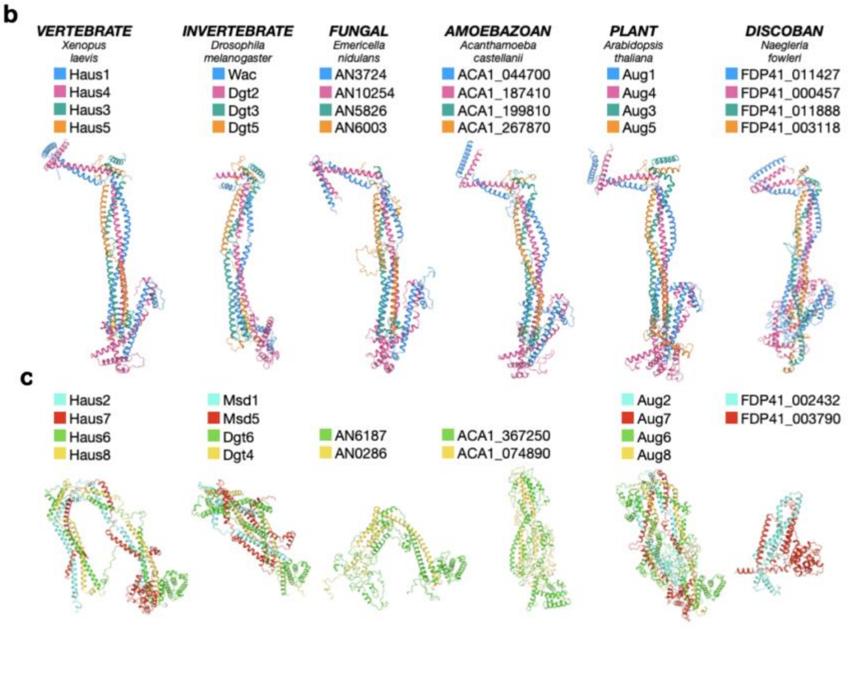

<u>Supplementary Figure 6: Conservation of the augmin complex across the eukaryotic kingdom</u>

a. Results of sequence-based search for Haus6 orthologs across eukaryotes, with species listed on left and ortholog UniProt ID on right (species where no ortholog was detected are marked with N/A). Species selected for further modeling are marked in boldface.

b. Augmin subunits in six representative eukaryotic species were identified either by prior independent work—*E. nidulans*[2] and *A. thaliana*[3]—or by bioinformatic search of the assembled predicted proteome. Complete T-III^{core} complexes and partial or complete T-II complexes were modeled by AlphaFold2-Multimer.

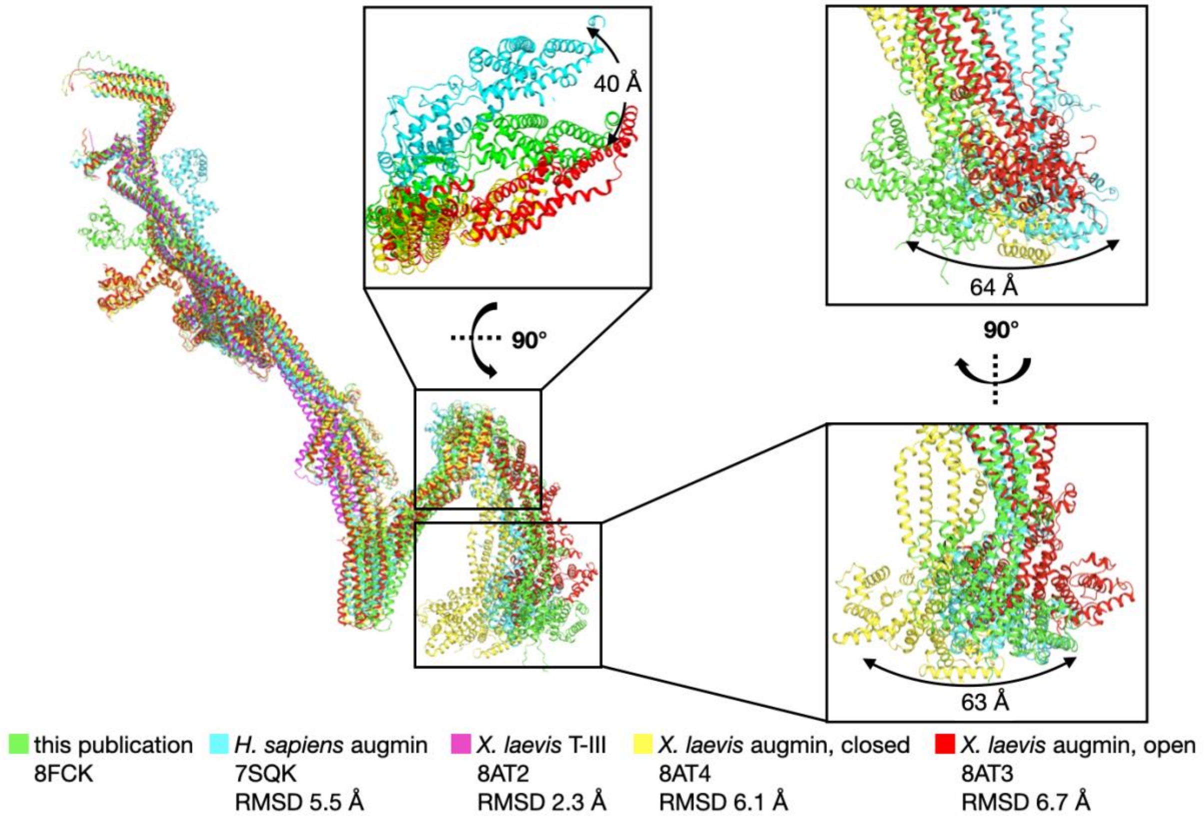

Supplementary Figure 7: Comparison of recently published structures of the augmin complex

The structures of four recently published augmin structures (*H. sapiens* augmin[4], cyan; *X. laevis* T-III39, magenta[5]; closed *X. laevis* augmin, yellow[5]; open *X. laevis* augmin, red[5]) drawn from the PDB coordinates listed below, are shown superimposed with our model of *X. laevis* augmin (green). The Cα superposition RMSD with our *X. laevis* augmin structure is shown below the PDB coordinates. Insets are given to show major points of divergence between T-II of the structures, as well as the maximum displacement between the models in the inset.

Supplementary Table 1: Cryo-EM data collection statistics

	dataset1	dataset2
Krios location	WUSTL	CWRU
Direct Detector	K2	K3
Cs (spherical aberration) (mm)	0.01[a]	2.7
Magnification	105,000 X	64,000 X
Pixel size (Å)	1.1	1.37
Total electron dose (e^-/Å^2)	84.3	66
No. of images	930	2,350

[a]The Titan Krios microscope at WUSTL is equipped with a Cs corrector.

Supplementary Table 2: Top DALI-AlphaFold2 *H. sapiens* database hits for augmin subunits
The Z-score is DALI's internal optimized similarity score, RMSD is the Cα root-mean-square displacement, and %ID is the pairwise sequence identity. Augmin subunits are highlighted in green, and NN-CH fold proteins in yellow.

**Z-score**	**RMSD**	**%ID**	**Protein name**
			**HAUS1**
7.2	29.9	5	C-TYPE LECTIN DOMAIN FAMILY 10 MEMBER A
7.1	14.8	6	TROPONIN T, SLOW SKELETAL MUSCLE
7.1	14.5	12	HAUS AUGMIN-LIKE COMPLEX SUBUNIT 1
7.0	39.6	8	INTRAFLAGELLAR TRANSPORT PROTEIN 74 HOMOLOG
7.0	42.0	7	TROPONIN T, CARDIAC MUSCLE
6.8	6.2	13	DACHSHUND HOMOLOG 2
6.8	11.4	5	PUTATIVE TRANSMEMBRANE PROTEIN CXORF1
6.7	42.3	6	TRANSMEMBRANE AND COILED-COIL DOMAINS PROTEIN 2
6.7	4.5	11	SEPTIN-8
6.7	50.0	6	JANUS KINASE AND MICROTUBULE-INTERACTING PROTEIN
			**HAUS2**
5.7	15.8	9	TROPONIN T, SLOW SKELETAL MUSCLE
5.5	19.5	8	SEPTIN-11
5.4	11.6	13	C-TYPE LECTIN DOMAIN FAMILY 10 MEMBER A
5.4	54.1	8	DYSBINDIN
5.4	25.8	5	POTE ANKYRIN DOMAIN FAMILY MEMBER D
5.4	24.2	4	RAB-3A-INTERACTING PROTEIN
5.3	24.5	10	MAJOR VAULT PROTEIN
5.3	42.8	8	CELL DIVISION CYCLE 5-LIKE PROTEIN
5.3	23.9	8	TROPONIN T, CARDIAC MUSCLE
5.3	23.3	9	TNF RECEPTOR-ASSOCIATED FACTOR 1
			**HAUS3**

9.6	39.0	34	HAUS AUGMIN-LIKE COMPLEX SUBUNIT 3
7.5	33.6	7	DYSTROPHIN
6.9	27.8	8	DYNEIN HEAVY CHAIN 5, AXONEMAL
6.8	69.3	5	KERATIN, TYPE I CYTOSKELETAL 28
6.8	73.1	9	WD REPEAT-CONTAINING PROTEIN 87
6.7	42.0	12	C-TYPE LECTIN DOMAIN FAMILY 3 MEMBER A
6.7	62.6	7	KERATIN, TYPE II CYTOSKELETAL 8
6.7	57.0	5	TRANSCRIPTIONAL REPRESSOR P66-BETA
6.6	75.2	7	KERATIN, TYPE II CUTICULAR HB6
6.6	12.0	8	CYTOPLASMIC DYNEIN 2 HEAVY CHAIN 1
**HAUS4**			
11.2	6.6	41	HAUS AUGMIN-LIKE COMPLEX SUBUNIT 4
5.9	16.5	9	LARGE NEUTRAL AMINO ACIDS TRANSPORTER SMALL SUBUNIT
5.5	20.8	5	E3 UBIQUITIN-PROTEIN LIGASE RNF213
5.4	23.9	8	E3 UBIQUITIN-PROTEIN LIGASE RNF213
5.1	16.1	9	ANKYRIN REPEAT DOMAIN-CONTAINING PROTEIN 24
5.1	34.8	5	E3 UBIQUITIN-PROTEIN LIGASE RNF213
5.1	14.7	6	E3 UBIQUITIN-PROTEIN LIGASE RNF213
5.0	20.8	11	PEPTIDE CHAIN RELEASE FACTOR 1, MITOCHONDRIAL
5.0	14.9	9	KERATIN, TYPE I CYTOSKELETAL 19
5.0	9.3	10	MICOS COMPLEX SUBUNIT MIC25
**HAUS5**			
9.3	17.8	23	HAUS AUGMIN-LIKE COMPLEX SUBUNIT 5
5.2	60.3	4	TRIPARTITE MOTIF-CONTAINING PROTEIN 55
5.1	20.1	8	PROTEIN BROAD-MINDED
5.0	24.4	8	SERINE/THREONINE-PROTEIN PHOSPHATASE 2A

5.0	12.4	6	ATP-BINDING CASSETTE SUB-FAMILY A MEMBER 13
4.9	27.4	1	SERINE/THREONINE-PROTEIN PHOSPHATASE 2A
4.8	63.5	7	STRUCTURAL MAINTENANCE OF CHROMOSOMES PROTEIN 1A
4.8	62.3	6	PUTATIVE RNA-BINDING PROTEIN LUC7-LIKE 2
4.8	59.7	8	THO COMPLEX SUBUNIT 5 HOMOLOG
4.8	14.6	6	PUTATIVE RNA-BINDING PROTEIN LUC7-LIKE 1
**HAUS6**			
25	10.2	40	HAUS AUGMIN-LIKE COMPLEX SUBUNIT 6
12.4	33.2	10	CLUSTERIN-ASSOCIATED PROTEIN 1
10.4	14.2	12	KINETOCHORE PROTEIN NDC80 HOMOLOG
10.3	30.6	13	INTRAFLAGELLAR TRANSPORT PROTEIN 57 HOMOLOG
10.2	24.7	6	HAUS AUGMIN-LIKE COMPLEX SUBUNIT 7
10	16.3	9	COILED-COIL DOMAIN-CONTAINING PROTEIN 22
9.2	20.7	7	KINETOCHORE PROTEIN NUF2
9.1	6.8	11	CENTROSOMAL PROTEIN OF 44 KDA
8.8	24	11	PROTEIN FAM98C
8.8	10.7	7	TRAF3-INTERACTING PROTEIN 1
8.8	28.2	10	PROTEIN FAM98B
8.4	23.8	10	SPERM FLAGELLAR PROTEIN 2
7.9	15.3	8	SPERM FLAGELLAR PROTEIN 1
7.9	22.9	7	COILED-COIL DOMAIN-CONTAINING PROTEIN 93
**HAUS7**			
19.9	25.6	33	HAUS AUGMIN-LIKE COMPLEX SUBUNIT 7
10.7	24.7	12	COILED-COIL DOMAIN-CONTAINING PROTEIN 22
9.7	22.3	9	CLUSTERIN-ASSOCIATED PROTEIN 1
9.2	12.5	9	HAUS AUGMIN-LIKE COMPLEX SUBUNIT 6
8.5	17.5	10	PROTEIN FAM98B

8.5	19.1	8	PROTEIN FAM98C
8.3	19.7	10	INTRAFLAGELLAR TRANSPORT PROTEIN 81 HOMOLOG
8.3	22.5	10	KINETOCHORE PROTEIN NDC80 HOMOLOG
8.3	30.9	9	PROTEIN FAM98A
8.2	8.3	13	CALMIN
**HAUS8**			
6.2	25.5	5	NEUROBLASTOMA BREAKPOINT FAMILY MEMBER 3
6.1	13.3	5	MERLIN
6.1	56.6	6	RAB GTPASE-BINDING EFFECTOR PROTEIN 2
6.0	62.7	13	PROTEIN SOGA1
6.0	14.4	3	PROHIBITIN-2
5.9	42.2	8	SYNAPTIC VESICLE GLYCOPROTEIN 2C
5.9	25.8	9	TRIPARTITE MOTIF-CONTAINING 51G, PSEUDOGENE
5.8	20.7	6	PLASMALEMMA VESICLE-ASSOCIATED PROTEIN
5.8	35.0	6	RIB43A-LIKE WITH COILED-COILS PROTEIN 1
5.7	40.0	10	MITOCHONDRIA-EATING PROTEIN

Supplementary Table 3: High-confidence *D. melanogaster* crosslinks and their predicted distances

BS3-crosslinks between the indicated residues are listed, along with their predicted distance in the AlphaFold2-Multimer *D. melanogaster* augmin model (Figure S5). Crosslinks are derived from[1]. Disallowed cross-links (greater than 24 Å) are highlighted in yellow.

**Subunit 1**	**Residue 1**	**Subunit 2**	**Residue 2**	**Distance**
Dgt2	Lys-193	Dgt3	Lys-508	18 Å
Dgt2	Lys-55	Dgt5	Lys-154	12 Å
Dgt2	Lys-214	Dgt5	Lys-653	9 Å
Dgt2	Lys-193	Wac	Lys-132	18 Å
Dgt2	Lys-214	Wac	Lys-146	21 Å
Dgt2	Lys-214	Wac	Lys-143	17 Å
Dgt2	Lys-193	Wac	Lys-140	19 Å
Dgt2	Lys-217	Wac	Lys-143	16 Å
Dgt2	Lys-193	Wac	Lys-143	27 Å
Dgt2	Lys-217	Wac	Lys-146	13 Å
Dgt3	Lys-72	Dgt5	Lys-73	16 Å
Dgt3	Lys-72	Dgt5	Lys-71	6 Å
Dgt3	Lys-119	Dgt5	Lys-124	6 Å
Dgt3	Lys-119	Dgt5	Lys-116	8 Å
Dgt3	Lys-249	Dgt5	Lys-286	7 Å
Dgt3	Lys-324	Dgt5	Lys-383	12 A
Dgt3	Lys-322	Dgt5	Lys-378	4 Å
Dgt3	Ser-165	Msd1	Lys-113	120 Å
Dgt3	Lys-508	Wac	Lys-132	28 Å
Dgt4	Lys-97	Msd5	Lys-174	20 Å
Dgt5	Lys-625	Wac	Lys-132	10 Å
Dgt5	Ser-606	Wac	Lys-132	30 Å
Dgt6	Lys-270	Msd1	Lys-78	8 Å

Dgt6	Lys-71	Msd5	Lys-87	21 Å
Dgt6	Lys-143	Msd5	Lys-87	15 Å
Dgt6	Lys-71	Msd5	Lys-86	23 Å

SUPPLEMENTARY REFERENCES

1. Chen JWC*, et al.* Cross-linking mass spectrometry identifies new interfaces of Augmin required to localise the gamma-tubulin ring complex to the mitotic spindle. *Biol Open* **6**, 654-663 (2017).

2. Edzuka T, Yamada L, Kanamaru K, Sawada H, Goshima G. Identification of the augmin complex in the filamentous fungus Aspergillus nidulans. *PLoS One* **9**, e101471 (2014).

3. Ho CM*, et al.* Augmin plays a critical role in organizing the spindle and phragmoplast microtubule arrays in Arabidopsis. *Plant Cell* **23**, 2606-2618 (2011).

4. Gabel CA*, et al.* Molecular architecture of the augmin complex. *Nat Commun* **13**, 5449 (2022).

5. Zupa E*, et al.* The augmin complex architecture reveals structural insights into microtubule branching. *Nat Commun* **13**, 5635 (2022).
