# Supplementary material for: Integrated model of the vertebrate augmin complex
Source: Nat Commun. 2023 Apr 13;14:2072. doi: 10.1038/s41467-023-37519-4 (PMC10102177; doi:10.1038/s41467-023-37519-4)
Supplement: Supplementary file 3 — Reporting Summary [file 41467_2023_37519_MOESM3_ESM.pdf]

nature portfolio

Corresponding author(s): Sabine Petry

Last updated by author(s): Feb 21, 2023

Reporting Summary

Nature Portfolio wishes to improve the reproducibility of the work that we publish. This form provides structure for consistency and transparency in reporting. For further information on Nature Portfolio policies, see our Editorial Policies and the Editorial Policy Checklist.

Statistics

For all statistical analyses, confirm that the following items are present in the figure legend, table legend, main text, or Methods section.

n/a	Confirmed	
☐	☒	The exact sample size (*n*) for each experimental group/condition, given as a discrete number and unit of measurement
☐	☒	A statement on whether measurements were taken from distinct samples or whether the same sample was measured repeatedly
☒	☐	The statistical test(s) used AND whether they are one- or two-sided<br>*Only common tests should be described solely by name; describe more complex techniques in the Methods section.*
☒	☐	A description of all covariates tested
☒	☐	A description of any assumptions or corrections, such as tests of normality and adjustment for multiple comparisons
☐	☒	A full description of the statistical parameters including central tendency (e.g. means) or other basic estimates (e.g. regression coefficient) AND variation (e.g. standard deviation) or associated estimates of uncertainty (e.g. confidence intervals)
☒	☐	For null hypothesis testing, the test statistic (e.g. *F*, *t*, *r*) with confidence intervals, effect sizes, degrees of freedom and *P* value noted<br>*Give P values as exact values whenever suitable.*
☒	☐	For Bayesian analysis, information on the choice of priors and Markov chain Monte Carlo settings
☒	☐	For hierarchical and complex designs, identification of the appropriate level for tests and full reporting of outcomes
☒	☐	Estimates of effect sizes (e.g. Cohen's *d*, Pearson's *r*), indicating how they were calculated

Our web collection on statistics for biologists contains articles on many of the points above.

Software and code

Policy information about availability of computer code

Data collection	NIS-Elements was used to acquire TIRF data. TIA was used to collect negative stain data. EPU was used to acquire cryo-EM data.
Data analysis	ImageJ 2.3.0/1.53f was used to process TIRF data. Relion 3.0.6 was used to process negative stain data. CryoSPARC v3.3.2+220824 was used to process cryo-EM data.

For manuscripts utilizing custom algorithms or software that are central to the research but not yet described in published literature, software must be made available to editors and reviewers. We strongly encourage code deposition in a community repository (e.g. GitHub). See the Nature Portfolio guidelines for submitting code & software for further information.

Data

Policy information about availability of data

All manuscripts must include a data availability statement. This statement should provide the following information, where applicable:

- Accession codes, unique identifiers, or web links for publicly available datasets
- A description of any restrictions on data availability
- For clinical datasets or third party data, please ensure that the statement adheres to our policy

The reconstructed electron density map can be obtained from the EMDB using accession code EMD-28981. The rigid-body fit structural model associated with this map can be obtained from the PDB using accession code 8FCK.

Human research participants

Policy information about studies involving human research participants and Sex and Gender in Research.

Reporting on sex and gender	N/A
Population characteristics	N/A
Recruitment	N/A
Ethics oversight	N/A

Note that full information on the approval of the study protocol must also be provided in the manuscript.

Field-specific reporting

Please select the one below that is the best fit for your research. If you are not sure, read the appropriate sections before making your selection.

[x] Life sciences [ ] Behavioural & social sciences [ ] Ecological, evolutionary & environmental sciences

For a reference copy of the document with all sections, see nature.com/documents/nr-reporting-summary-flat.pdf

Life sciences study design

All studies must disclose on these points even when the disclosure is negative.

Sample size	For negative stain and cryo-EM data collection, sample size (ie number of particles) was determined by intended final structural resolution. Sample sizes are listed in the Methods section and Supplementary Figure 2a.
Data exclusions	For negative stain and cryo-EM data processing, particles were excluded based on automated sorting into low resolution 2D and 3D classes. For cryo-EM, data exclusion is diagrammed in Supplementary Figure 2a.
Replication	For in vitro reconstitution of branching MT nucleation, experiments were replicated twice (technical replicates performed on different days with different batches of augmin and γ-TuRC). For cryo-EM, two different data sets were collected on the same sample, as detailed in Supplementary Table 1. EM samples were not performed in technical replicate.
Randomization	For determination of cryo-EM resolution, particles were randomly assigned to half-map A or B by cryoSPARC and processed independently.
Blinding	It is standard in the field of structural biology and biochemistry that blinding is not relevant.

Reporting for specific materials, systems and methods

We require information from authors about some types of materials, experimental systems and methods used in many studies. Here, indicate whether each material, system or method listed is relevant to your study. If you are not sure if a list item applies to your research, read the appropriate section before selecting a response.

Materials & experimental systems

n/a	Involved in the study	
[x]	[ ]	Antibodies
[ ]	[x]	Eukaryotic cell lines
[x]	[ ]	Palaeontology and archaeology
[ ]	[x]	Animals and other organisms
[x]	[ ]	Clinical data
[x]	[ ]	Dual use research of concern

Methods

n/a	Involved in the study	
[x]	[ ]	ChIP-seq
[x]	[ ]	Flow cytometry
[x]	[ ]	MRI-based neuroimaging

Eukaryotic cell lines

Policy information about cell lines and Sex and Gender in Research

Cell line source(s)	Sf9 cells were purchased from ATCC
Authentication	The cell line was not authenticated subsequent to receipt
Mycoplasma contamination	The cell line was not tested for mycoplasma contamination subsequent to receipt
Commonly misidentified lines (See ICLAC register)	No commonly misidentified lines were used in this research

Animals and other research organisms

Policy information about studies involving animals; ARRIVE guidelines recommended for reporting animal research, and Sex and Gender in Research

Laboratory animals	Adult, female Xenopus laevis frogs were used in the study
Wild animals	The study did not involve wild animals
Reporting on sex	Only female frogs were used, as unfertilized Xenopus laevis eggs were required for generation of meiotic Xenopus egg extract
Field-collected samples	The study did not involve samples collected in the field
Ethics oversight	The Princeton University Institutional Animal Care and Use Committee provided guidance and approved the use of Xenopus laevis frogs for this study (protocol number 1941).

Note that full information on the approval of the study protocol must also be provided in the manuscript.
